# Genetics and Epigenetics in Asthma

**DOI:** 10.3390/ijms22052412

**Published:** 2021-02-27

**Authors:** Polyxeni Ntontsi, Andreas Photiades, Eleftherios Zervas, Georgina Xanthou, Konstantinos Samitas

**Affiliations:** 1 7th Respiratory Medicine Department and Asthma Center, Athens Chest Hospital “Sotiria”, 11527 Athens, Greece; xenia-1990@hotmail.com (P.N.); anpho81@hotmail.com (A.P.); lefzervas@yahoo.gr (E.Z.); 2 Cellular Immunology Laboratory, Biomedical Research Foundation of the Academy of Athens, 11527 Athens, Greece; gxanthou@bioacademy.gr

**Keywords:** asthma, genetics, epigenetics, atopy, DNA methylation, GWAS

## Abstract

Asthma is one of the most common respiratory disease that affects both children and adults worldwide, with diverse phenotypes and underlying pathogenetic mechanisms poorly understood. As technology in genome sequencing progressed, scientific efforts were made to explain and predict asthma’s complexity and heterogeneity, and genome-wide association studies (GWAS) quickly became the preferred study method. Several gene markers and loci associated with asthma susceptibility, atopic and childhood-onset asthma were identified during the last few decades. Markers near the *ORMDL3/GSDMB* genes were associated with childhood-onset asthma, interleukin (IL)33 and *IL1RL1* SNPs were associated with atopic asthma, and the *Thymic Stromal Lymphopoietin (TSLP)* gene was identified as protective against the risk to TH2-asthma. The latest efforts and advances in identifying and decoding asthma susceptibility are focused on epigenetics, heritable characteristics that affect gene expression without altering DNA sequence, with DNA methylation being the most described mechanism. Other less studied epigenetic mechanisms include histone modifications and alterations of miR expression. Recent findings suggest that the DNA methylation pattern is tissue and cell-specific. Several studies attempt to describe DNA methylation of different types of cells and tissues of asthmatic patients that regulate airway remodeling, phagocytosis, and other lung functions in asthma. In this review, we attempt to briefly present the latest advancements in the field of genetics and mainly epigenetics concerning asthma susceptibility.

## 1. Introduction

Asthma is a complex, heterogenous but one of the most common respiratory diseases that affects both children and adults worldwide, with diverse phenotypes and underlying pathogenetic mechanisms poorly understood [1]. In the last decade, several genome-wide association studies (GWAS) have identified numerous genetic variants responsible for asthma susceptibility [2]. These mainly non-coding variants play a regulatory role in gene expression and asthma heritability [3]. Despite these results, genetics could not fully explain asthma development and are of limited value. The novel field of epigenetics has recently attracted the attention of researchers. Epigenetic changes such as DNA methylation, histone modifications, and microRNA expression have already been studied in several research projects, which could lead to a better understanding of the disease’s mechanisms [4]. In this review, we will present the latest advances of genetic and epigenetic studies in asthma susceptibility and discuss further implications. Moreover, we present current knowledge on DNA methylation of different types of immune cells in peripheral blood as well as the alterations in gene methylation in nasal and bronchial epithelium of asthmatic patients.

## 2. Genetics in Asthma

Nowadays, it is widely accepted that asthma susceptibility has a strong genetic component, as shown by multiple studies. Identifying the specific genetic loci associated with asthma and uncovering the molecular mechanism in which those loci affect the risk of developing asthma will help the scientific community unravel and better understand the biological pathways implicated in asthma pathogenesis. The first real scientific effort to pinpoint the association between specific genes and asthma susceptibility began with the first publications of human genome sequencing. The two main approaches used are linkage studies and GWAS. Linkage studies aim to identify DNA patterns that correspond to the phenotype of asthma in family members with and without asthma, while GWAS use microarrays of single nucleotide polymorphism (SNP) chips. The aim is to identify DNA patterns or variations associated with asthma phenotypes or characteristics by comparing the complete DNA sequence of individuals with disease to ethnically matched individuals without disease [5,6]. As GWAS are large-scale population studies that identify polymorphisms in the human genome without the need to genotype the entire genome, they have become the preferred study method in asthma genetics throughout the last decades [7].

### 2.1. GWASs of Asthma

The first real effort to identify all important loci that correspond with asthma susceptibility was The Collaborative Study on the Genetics of Asthma (CSGA), published in 1997 [8]. This was the first study to consider the heterogeneity of asthma and the hypothesis that different genes regulate asthma characteristics in individuals of different racial backgrounds. The study analyzed asthmatic sib pairs from 3 different racial groups (African-Americans, Caucasians, and Hispanics) and identified six loci: *5p15* and *17p11.1—q ll.2* in African Americans; *11p15* and *19q 13* in Caucasians; *2q33* and *21q21* in Hispanics [8].

As technology in genome sequencing progressed, the number of GWAS studies rose exponentially, with various loci being associated with asthma susceptibility [9]. There are 8 meta-analyses concerning genetics in asthma susceptibility and the results are presented in Table 1. The genes most frequently studied and replicated are discussed in the text below.

#### 2.1.1. ORMDL3/GSDMB, PYHIN1

*ORMDL3* is the third member of a class of genes that encode transmembrane proteins anchored in the endoplasmic reticulum (ER) and regulate sphingolipid synthesis while *GSDMB* gene encodes a member of the gasdermin-domain containing protein family and is implicated in the regulation of apoptosis in epithelial cells. Markers near the *ORMDL3/GSDMB* genes on chromosome *17q21* were first associated with childhood-onset asthma by Moffatt MF et al. in a GWAS in a European sample in 2007 (*rs7216389* was the marker most strongly associated with disease) [18]. The GABRIEL Consortium, mainly composed of the same group of investigators, expanded the GWAS sample in 2010, confirming the association with the *17q21* locus and identifying five additional genes (*IL33* and *IL1RL1*, *IL18R1*, *SMAD3*, and *IL2RB*) [19]. The *ORMDL3/GSDMB* locus association was replicated in the EVE Consortium in the United States. However, later studies failed to replicate the most significantly associated SNPs among African ancestry subjects [20,21,22,23]. On the other hand, the EVE Consortium identified a susceptibility locus for asthma in the *PYHIN1* gene on chromosome 1q23 (pyrin and HIN domain family member 1; a major mediator of the tumor suppressor activity of IFN in breast cancer cells) that was unique in individuals of African descent with the marker *rs1102000* being most strongly associated with asthma [20]. This constitutes the first indication that ancestry-specific associations also contribute to the complex genetic architecture of asthma.

#### 2.1.2. Association between Asthma Risk Loci and Immune Cell Enhancer Marks (IL-33, IL1RL1, TSLP, IL33)

The EVE Consortium replicated the association between asthma susceptibility and SNPs near *IL33* (*rs2381416*) and *IL1RL1* (*rs10173081*) [20], although later studies showed a strong association with atopic asthma but not with non-atopic asthma [24], while also identifying a susceptibility locus near the *TSLP* gene (an epithelial-cell-derived cytokine that regulates allergic inflammation), where a single SNP (*rs1837253*) showed to be protective against the risk for TH2-asthma. This association led to the development of anti-TSLP agents to treat TH2-asthma [20].

In a meta-analysis of GWASs by Demenais et al. in 2018, a susceptibility locus (*rs20541*) near *IL13*, *RAD50*, and *IL4* genes associated with Th2-asthma phenotype was identified. The critical role of IL-13 in the IgE production and the eosinophilic pathway, along with the association between IL-13 and asthma severity, led to a shift in focus of the pharmaceutical society to the development of anti-IL-13 agents to treat asthma [16].

#### 2.1.3. GWASs of Different Racial Populations

Demenais et al. combined data of European, African, Japanese, and Latino populations included in the Trans-National Asthma Genetic Consortium (TAGC), and identified 18 genome-wide significant loci, including nearly all preciously reported loci. The meta-analysis identified as the 5 most significant loci on chromosomes *17q12-21* near *PGAP3* and *ERBB2*, *6p21.32* near *HLA-122 DRB1/-DQA1*, *5q22.1* near *TSLP*, *2q12* near *IL1RL1/IL18R*, and *9p24.1* near *IL33* in both the whole sample and in the subset that included only subjects with early-onset asthma [16]. In the latter subset, the significant variants at the *17q12-21* locus were nearest to *GSDMB* and *ORMDL3* [19,20,25], findings that were in agreement with earlier GWASs. The meta-analysis also showed large overlaps in genetic variants with autoimmune and inflammatory diseases [16], but also with diseases that are characterized as allergic or immune-mediated [26].

#### 2.1.4. GWASs of Asthma Age of Onset

Early-onset asthma and adult-onset asthma represent distinct asthma phenotypes with differences regarding severity, remission of symptoms, comorbidities, and sex ratios [27,28]. The Pividori et al. [29] and the Ferreira et al. studies [30] used genotype and phenotype data from the UK Biobank and performed several GWASs: subjects with asthma, including childhood-onset cases (onset before age 12) and adult-onset cases (onset between ages 26 and 65), subjects without asthma (controls; older than age 38) and two GWASs (childhood and adult-onset cases, respectively) in an effort to describe novel loci (additional to the highly replicated locus at *17q12-21*) associated with various ages of onset.

The Pividori et al. study identified 61 independent asthma loci, out of which 23 were childhood-onset specific and one was adult-onset specific, while 37 were shared between the subsets. Interestingly, 28 of the 61 identified loci had not been previously described as associated with asthma in previous studies. The study also suggests that there is a greater role for genetic risk factors in childhood-onset asthma than in adult-onset asthma, and as a more heterogeneous condition, the role for non-genetic risk factors appears to be of greater significance [29].

The Ferreira et al. study also identified 98 independent genetic associations for childhood-onset asthma and 34 for adult-onset asthma, as well as 109 likely target genes of the risk variants. As with the Pividori et al. study, 28 of the 132 replicated variants loci had not been described in previous studies as associated with asthma. Ferreira et al. also confirmed the greater role of genetic factors of childhood-onset asthma in contrast with heterogeneity of adult-onset asthma, where genetic risk factors are shared with environmental factors and comorbidities [30].

### 2.2. Whole Genome Sequencing

One of the major limitations of GWAS is the observation that most SNPs associated with asthma susceptibility are located in non-coding regions of the genome. This was also confirmed in the TAGC meta-analysis [20]. The most optimal way to resolve this issue is by using whole-genome sequencing (WGS), which seems to be superior in accurately determining genotypes of copy number variants (CNVs) and low-frequency variants missed by genome-wide association studies. In a study published by Hoglund et al. 72 inflammatory biomarkers were analyzed and 18 novel associations were identified when using GWAS approach on WGS data, that were not detected when analyzing the same biomarkers with genotyped or imputed SNPs. This study suggests that we can enhance the power and accuracy of GWAS when using WGS data by having the ability to identify quantitative trait loci and nucleotides (QTNs) [31,32]. A few programs, including the Consortium of Asthma in African Populations of the Americas (CAAPA) and the Trans-Omics for Precision Medicine program (TOPMed) have begun using WGS, and several CNVs, structural variants, and rare coding variants have already been identified [33,34,35].

## 3. Epigenetics in Asthma

Epigenetics are heritable characteristics that affect gene expression without altering DNA sequence in contrast to genetics [36]. Prenatal environmental factors such as maternal smoking and other factors after birth such as traffic-related or air pollution, nutrients and drugs could be the triggering factors of epigenetic changes [37]. Epigenetic modifications occur during prenatal development, early childhood and adolescence as these are the periods during life that people are susceptible to several asthma triggers [38]. Apart from the skin, the respiratory tract is directly exposed to the external environment and as a result buccal mucosa, nasal and bronchial epithelium are firstly subjected to epigenetic alterations [38]. Moreover, approximately 17 studies and meta-analyses have identified the methylation patterns of immune cells in whole and peripheral blood of asthmatic patients [39].

DNA methylation, post-translational histone modifications and microRNA expression are the most common epigenetic mechanisms identified and have a regulatory role in immune responses and gene expression in asthma. Moreover, it is important to note that epigenome is distinct for each cell type [4].

Several studies like epigenome-wide association studies (EWAS), experimental and observational projects, have been conducted and published the previous years in an effort to clarify the interaction between environment and specific immune pathways that undergo epigenetic regulation [2,37].

### 3.1. DNA Methylation

Among epigenetic mechanisms, the most described in literature is CpG-DNA methylation which refers to the addition of a methyl group to a cytosine in a CpG dinucleotide and thus, cytosine is converted to 5′-methylcytosine [38]. The methylation is catalysed by DNA methyltransferases (DNMT1, DNMT2 and DNMT3) [39]. DNA methylation in CpG islets near the promoter region of the genes usually leads to the repression of transcription, whereas hypomethylation has as a result the upregulation of gene expression [39].

The EWAS are mainly cross-sectional studies and for that reason, they cannot distinguish whether the epigenetic change precedes the development of the disease and has a causal relationship or is just a consequence [2]. Notably, the EWAS performed in nasal epithelium present higher degree of reproducibility in other cohorts than EWAS in blood. According to current data, blood-based EWAS is mainly representative of the eosinophilic part of the disease, while EWAS of nasal epithelium reflect the methylation changes and dysregulation of airway epithelial cells in the respiratory tract [2]. All these recent findings imply that the methylation pattern is cell and tissue-specific [4]. On the other hand, a significant overlap of methylation changes in nasal cells with those observed in bronchial epithelial cells, that are difficult to obtain especially in children, has been noted [2]. Some genes were common in both blood-based EWAS and in nasal epithelium such as *ACOT7*, *EPX*, *GJA4*, and *METTL1* [2]. These results lead to the conclusion that apart from cell-specificity of CpG sites of methylation, there is also cross-tissue epigenetic effect [40].

DNA Methylation patterns are discussed below, divided in two separate sections including blood immune cells and airway epithelial cells.

#### 3.1.1. Immune Cells

##### Granulocytes

The best studied type of cells is eosinophils. The target genes of eosinophils for epigenetic modification implicated in the pathophysiology of asthma are hypomethylated and this fact affects the immune functions of eosinophils, the interaction with other PBMCs and certain lung functions [40,41]. For instance, genes implicated in airway remodeling (*COL15A1*, *RB1*, *FOXP1*, *CCDC19*), surfactant secretion (*ACOT7*, *PPT2*) and nitric oxide production in airways (*ACP5*), as well as genes associated with cytokine production and signaling (*IL5RA*, *DICER1*) and phagocytosis in blood (*SERPINC1*) are characterized by decreased methylation in asthmatic subjects [39].

Few data are available for other granulocytic cells. For instance, hypomethylation of *histidine decarboxylase* gene (*HDC*) of basophils and mast cells is associated with increased histamine formation, a significant inflammatory mediator in allergic asthma [42].

##### Mononuclear Cells

Blood monocytes differentiate into tissue macrophages in vitro by demethylating phagocytic genes by TET enzymes [43]. A study has been conducted in order to assess alterations in DNA methylation of blood monocytes in individuals with distinct asthma phenotypes (eosinophilic, neutrophilic and pauci-granulocytic), and nine common loci, all hypermethylated, were discovered [44]. These nine loci, for instance *NRG1*, *SYNM*, *TBX5*, FAM19A4, are all involved in airway remodeling and disruption as well as macrophage function [44]. On the other hand, several methylation changes were found and correlated with specific asthma inflammatory phenotypes and could be used as biomarkers to diagnose or guide treatment in the future [44]. Naïve CD4+ T-cells differentiate into Th1 cells by methylation of the promoter region of IL-4 and demethylation of CpG sites within the interferon gamma (IFN-γ) gene, while differentiation into Th2 cells is associated with demethylation in IL-4 and IL-13 promoters that allows binding of transcription factors STAT6 and GATA3 [45,46]. However, Th2 cells could reactivate the production of IFN-γ by demethylating the gene promoter and this fact proves the epigenetic plasticity of the production of IFN-γ in these cells mediated by STAT4 and T-box expressed in T cells (T-bet) [47]. Moreover, demethylation of the *forkhead box protein 3* (*FOXP3*) gene is required so as to activate the suppressive role of regulatory T-cells [48,49]. High levels of air pollution lead to hypermethylation of *FOXP3* in peripheral blood Treg cells and their functional impairment in asthmatic individuals [50].

Th17 cells, a third subset of CD4+ T-cells, are crucial for the defense of the immune system against fungi and extracellular bacteria and are also implicated in asthma pathogenesis [37]. Mukasa et al. demonstrated that Th17 cell lineage is subjected to epigenetic plasticity through the remodeling of the chromatin structure [51]. DNA methylation is also important for the differentiation of naive CD8+ T-cells into effector cells in non-pathological conditions, while studies in asthma epigenetics need to be designed [52].

In an experimental study, house dust mite allergic subjects had a different epigenome of B+ cells from non-allergic with 451 differentially methylated loci [53]. A subset of lymphocytes B produces IgE in allergic asthma and the most important genes associated with antigen presentation and IL-4 signaling such as *CCDC80*, *DAPK3*, *LOXL1*, *PROC*, *FUCA2*, *SP100*, *ITCH*, present increased methylation [53]. Furthermore, the promoter of *CYP26A1* gene involved in retinoic acid clearance presents hypermethylation in B cells of asthmatic individuals [53]. No data available on DNA methylation of dendritic and natural killer cells of asthmatic patients [39].

#### 3.1.2. Airway Cells

The respiratory tract is the target of the immune system in bronchial asthma and consists of airway epithelial cells, goblet cells, fibroblasts and airway smooth muscle cells [54]. Nasal epithelial cells are more easily accessible than the bronchial epithelial cells and less invasive techniques are needed to obtain samples from nasal cells [39]. For these reasons, the information concerning DNA methylation of airway cells in childhood asthma mainly derives from studies of nasal epithelium. Forno et al. defined a panel of 30 CpGs in their cohort of 483 school-aged children in Puerto Rico that could be used to predict atopy and atopic asthma [55]. Moreover, no studies are available from sputum samples and only one small study have been performed in saliva samples of atopic children [56].

### 3.2. Histone Modifications

DNA is packaged by core histones to form an organized chromatin structure. The core histones consist of H2A, H2B, H3 and H4 [57]. Post-translational histone modifications, such as acetylation, methylation, phosphorylation, ubiquitination, SUMOylation, and ADP-ribosylation on the tails of core histones, represent another important classical epigenetic mechanism in numerous diseases including bronchial asthma, although it is not as widely studied as DNA methylation [57]. Histone acetylation by histone acetyltransferase (HAT) usually results in a loose structure of chromatin, easily accessible to transcription factors that leads to the activation of gene expression. On the other hand, histone deacetylation by histone deacetylase (HDAC) results in gene silencing. HAT activity is elevated in biopsies in both adults [58] and in children [59] and the HAT/HDAC ratio alters according to asthma severity. HDAC inhibitors, particularly HDAC2, are potential targeted therapies against asthma but the results of the clinical trials so far are controversial [57,60,61].

HDACs are implicated in T-cells development and their inhibition could lead to allergic airway diseases. Moreover, defective HDAC2 activity is found in corticosteroid-insensitive severe asthma phenotypes [62,63] and mice with HDAC1- deficient T-cells present increased eosinophil recruitment and Th2 cytokine production [61]. HDAC1 plays an important role in airway epithelial repair and remodeling and is found increased in severe asthma characterized by airway remodeling compared to mild asthma [64].

Histone H3 lysine 18 (H3K18) acetylation increases the expression of EGFR and STAT6 as it is expected to be in the epithelium of asthmatic patients [65].

Moreover, histone methylation is associated with CD4+ T-cell differentiation [4]. Histone H3 lysine 4 trimethylation (H3K4me3) is linked to increased transcription of both IFN-γ and IL-4 [66]. H3K27me3 can have various functions on gene transcription based on the location of the histone compared with the location of the gene [4]. For instance, H3K27me3 inhibits the production of IL-4 in TH1 cells whereas in TH2 cells, H3K27me3 represses IFN-γ [67]. A decrease in H3K27me3 by JMJD2D demethylase at the promoter of the VEGF gene has been observed in asthmatic airway smooth muscle cells [68]. The location of H3K4me3 and H3K27me3 around genes of dendritic cells contributes to the determination of their inflammatory state and their transition to antigen-presenting cells [69].

It is widely acceptable that post-translational histone modifications play a key role in asthma pathogenesis and more studies need to be conducted to clearly understand their contribution to immune responses, the interaction with transcription factors and between different modifications.

### 3.3. MicroRNA (miR) Expression

MiRNAs are approximately 20 nucleotides long non-coding, highly conserved RNA that act as regulators of gene expression by controlling the translation of as many as 60% of mRNAs via mRNA destabilization [57,70]. There are differences in miR expression in asthma compared to healthy controls, mainly in a cell-specific manner [4]. MiRNAs, such as MiRNA *let-7f*, MiRNA-9, MiRNA-17-18-19-20-92, MiRNA-26a/b, MiRNA-27a/b, MiRNA-125b, MiRNA-155, polarize macrophages towards a pro-inflammatory M1 phenotype while miRNA *let-7a/b/c/d/e*, MiRNA-21, MiRNA-34, MiRNA-124, MiRNA-146a/b, MiRNA-223-3p, MiRNA-511-3p promote the polarization towards an anti-inflammatory M2 phenotype [71].

In specific, miRNA-19 is upregulated in the epithelium of severe asthmatic patients, targets TGFB2 mRNA, thus contributing to airway remodeling [72]. Furthermore, IL-13 induces the decrease of miRNA-34/449 in bronchial epithelial cells [73] and increases in vitro the expression of miR-21 and miR-126 [74]. MiR-21 and miR-126 are upregulated in asthmatic patients compared to control, especially in patients without treatment [74]. Moreover, miR-21 contributes to eosinophilic production and proliferation whereas miR-223 suppresses it [75]. Overexpression of miR-21 is associated with the in vitro differentiation of Th2 cells, while miR-27 and miR-128 reduce IL-4 and IL-5 production from CD4+ T cells [75,76]. MiRNA-21 is the best studied miR in asthma and its high levels are linked to IL-12p35 repression in serum of asthmatics and to steroid-insensitivity and that implies that it could be used as biomarker to monitor the response to steroid treatment [77].

Also, miR-15a, miR-15b, and miR-20a are downregulated in CD4+ T -cells from atopic pediatric patients with asthma in contrast to atopic and non-atopic subjects [78]. The downregulation of miR-15a leads to the overexpression of the vascular endothelial growth factor A (VEFGA) in sputum and serum of asthmatics [78].

In peripheral blood of asthmatics, miR-625-5p, miR-22-3p, and miR-513a-5p were downregulated compared to controls. The target genes of these miRNAs (*CBL*, *PPARGC1B*, and *ESR1*), inversely upregulated in blood, belong to the PI3K-AKT and nuclear factor κβ (NF-κβ) signaling pathways and could be related to the lower concentrations of IFN-γ, TNF-α, IL-12, and IL-10 in plasma [79]. In miR-155 -deficient mouse models, an increase in airway remodeling was observed [80] and in vitro miR-133a inhibition in human bronchial smooth muscle cells has as a consequence the upregulation of RhoA, a key protein in contractility of airway muscle cells [81]. The findings of miR studies are summarized in Table 2.

## 4. Conclusions

Asthma is a complex disease with multiple phenotypes based on underlying genetics and interactions with environmental factors. Using WGS on GWAS will help identify new genetic loci that predict asthma risk and severity. Epigenetic markers will be used to predict treatment response to novel therapies and guide treatment. Altered miRNA levels lead to modulation of cytokine signaling that orchestrates allergic airway inflammation and asthma. Thus, the development of miRNA targeted therapies could be a promising therapeutic measure. Future directions of clinical management are heading towards personalized therapy via pharmacogenetics and pharmacoepigenetics, although further studies and progress in these fields are required.

## Figures and Tables

**Table 1:** Presentation of the results of 8 significant meta-analyses concerning genetics in asthma susceptibility published on the GWAS Catalog and the loci associations that were identified.

Author and Study Accession	Discovery Sample Number and Ancestry	Replication Sample Number and Ancestry	Variant and Risk Allele	*p*-Value	Mapped Gene
Himes BE et al., 2010 GCST000768 [10]	-	-	0		
	
Himes BE et al., 2009 GCST000389 [11]	1205 European	1776 Hispanic or Latin American	*rs1588265-C*	3 × 10^−8^	*PDE4D*
		1776 European, Hispanic or Latin American	* *		* *
		5264 African American or Afro-Caribbean	* *		* *
		15339 European	* *		* *
Dahlin A et al., 2019 GCST007596 [12]	54543 European	26475 European	*rs17843604-?*	1 × 10^−18^	*HLA-DQB1, HLA-DQA1*
		NA NR	*rs9269080-?*	1 × 10^−8^	*HLA-DRB9*
			*rs1420101-T*	8 × 10^−15^	*IL1RL1, IL18R1*
Dahlin A et al., 2019 GCST007599 [12]	2526 African American or Afro-Caribbean	26475 European	*0*		
		NA NR			
Dahlin A et al., 2019 GCST007598 [12]	6227 Asian unspecified	NA NR	*0*		
		26475 European			
Dahlin A et al., 2019 GCST007597 [12]	5327 Hispanic or Latin American	26475 European	*0*		
		NA NR			
Barreto-Luis A et al., 2016 GCST003176 [13]	889 European	2206 European	*rs9866261-G*	1 × 10^−7^	*ADAMTS9*
			*rs10197862-?*	2 × 10^−6^	*IL18R1, IL1RL1*
Myers RA et al., 2014 GCST002445 [14]	3606 European	-	*rs2549003-G*	9 × 10^−7^	*AC116366.3*
	3015 African American or Afro-Caribbean		*rs17642749-G*	4 × 10^−7^	*RAB11FIP2, AL513324.1*
			*rs1012307-C*	8 × 10^−7^	*LINC01931, MMADHC-DT*
	2428 Hispanic or Latin American		*rs4673659-C*	9 × 10^−7^	*ERBB4*
			*rs2675724-A*	2 × 10^−7^	*C6orf118, AL136100.1*
			*rs9895098-C*	3 × 10^−7^	*RAP1GAP2*
Ferreira MA et al., 2011 GCST000910 [15]	2832 European	604 European	*rs6503525-C*	5 × 10^−7^	*AC090844.2*
		
Demenais F et al., 2018 GCST005212 [16]	5215 East Asian	-	*rs1348135-C*	9 × 10^−6^	*AL358875.1, AL355862.1*
	1398 Hispanic or Latin American		*rs10773588-G*	5 × 10^−6^	*NLRP9P1, TMEM132D*
			*rs10157802-G*	7 × 10^−6^	*LINC02238*
	8204 African unspecified, African American or Afro-Caribbean		*rs4268898-C*	2 × 10^−7^	*ITSN2*
			*rs9870718-C*	9 × 10^−6^	*STAC, RFC3P1*
			*rs10141207-A*	4 × 10^−6^	*RNU6-8, AL136298.2*
			*rs10924970-C*	3 × 10^−6^	*ARID4B*
	127,669 European		***rs992969*** *-A*	7 × 10^−20^	*GTF3AP1, IL33*
			*rs2952156-A*	2 × 10^−30^	*ERBB2*
			*rs20541-A*	5 × 10^−16^	*TH2LCRR, IL13*
			*rs11071558-A*	1 × 10^−9^	*RORA*
			*rs2325291-G*	2 × 10^−12^	*BACH2*
			*rs2589561-A*	4 × 10^−9^	*AC044784.1, LINC00709*
			*rs17806299-G*	3 × 10^−10^	*CLEC16A*
			*rs12543811-G*	1 × 10^−10^	*AC034114.2*
			*rs167769-T*	4 × 10^−9^	*STAT6*
			*rs17637472-A*	7 × 10^−9^	*AC091180.6*
			*rs7705042-A*	8 × 10^−9^	*NDFIP1*
			*rs1233578-G*	6 × 10^−7^	*RPSAP2, NOP56P1*
			*rs12634582-C*	9 × 10^−6^	*THRB*
			*rs2855812-T*	9 × 10^−12^	*MICB*
			*rs7927894-T*	2 × 10^−14^	*EMSY, AP001189.2*
			*rs2033784-G*	7 × 10^−15^	*SMAD3*
			*rs1420101-T*	4 × 10^−21^	*IL1RL1, IL18R1*
			*rs10455025-C*	9 × 10^−26^	*TSLP, AC010395.1*
			*rs9272346-A*	6 × 10^−24^	*HLA-DQA1*
			*rs4129267-C*	8 × 10^−7^	*IL6R*
			*rs4938096-T*	1 × 10^−6^	*AP002518.1*
			*rs16989837-T*	3 × 10^−6^	*AL035660.2, AL035660.1*
			*rs10233459-G*	4 × 10^−6^	*GLI3*
			*rs1122396-G*	2 × 10^−6^	*TMEM9*
			*rs10951405-T*	3 × 10^−6^	*BMPER*
			*rs3758697-A*	2 × 10^−6^	*ARHGAP42*
			*rs12542922-A*	2 × 10^−6^	*LINC00536*
			*rs2825968-T*	6 × 10^−6^	*LINC02573, LINC01683*
			*rs2073617-A*	5 × 10^−6^	*TNFRSF11B, RNU6-12P*
			*rs12521260-T*	3 × 10^−6^	*GDNF-AS1*
			*rs2352521-T*	5 × 10^−6^	*ADGRL4, AC104837.1*
			*rs6694672-G*	9 × 10^−6^	*AL139418.1, CFHR5*
			*rs6084352-G*	7 × 10^−6^	*C20orf194*
Demenais F et al., 2018 GCST005213 [16]	20,762 European	-	*rs4988958-T*	5 × 10^−13^	*IL1RL1, IL18R1*
	1398 Hispanic or Latin American		*rs1295685-A*	2 × 10^−9^	*IL13, TH2LCRR*
			*rs2596464-C*	1 × 10^−8^	*LINC01149, AL645933.5*
	5215 East Asian		*rs12551256-A*	3 × 10^−8^	*IL33*
			*rs8069176-G*	4 × 10^−26^	*ZPBP2, GSDMB*
Demenais F et al., 2018 GCST006862 [16]	127,669 European	-	*rs7705042-A*	9 × 10^−10^	*NDFIP1*
			*rs1233578-G*	5 × 10^−9^	*RPSAP2, NOP56P1*
			*rs2325291-A*	9 × 10^−13^	*BACH2*
			*rs167769-T*	6 × 10^−9^	*STAT6*
			*rs17637472-A*	3 × 10^−9^	*AC091180.6*
			*rs2596464-C*	2 × 10^−13^	*LINC01149, AL645933.5*
			*rs1663687-A*	2 × 10^−10^	*LINC00709, AC044784.1*
			*rs10957979-G*	2 × 10^−8^	*AC034114.2*
			*rs17806299-A*	2 × 10^−10^	*CLEC16A*
			*rs3771180-T*	2 × 10^−20^	*IL18R1, IL1RL1*
			*rs10455025-C*	2 × 10^−25^	*TSLP, AC010395.1*
			*rs20541-G*	1 × 10^−14^	*TH2LCRR, IL13*
			*rs9272346-A*	2 × 10^−28^	*HLA-DQA1*
			*rs992969-A*	4 × 10^−29^	*GTF3AP1, IL33*
			*rs2155219-T*	3 × 10^−15^	*EMSY, AP001189.2*
			*rs11071558-G*	8 × 10^−11^	*RORA*
			*rs17293632-T*	9 × 10^−16^	*SMAD3*
			*rs2305479-T*	3 × 10^−24^	*GSDMB*
			*rs2855812-T*	3 × 10^−10^	*MICB*
			*rs2844510-T*	2 × 10^−6^	*AL645933.5, LINC01149*
			*rs1420101-T*	9 × 10^−20^	*IL1RL1, IL18R1*
			*rs6893213-T*	8 × 10^−11^	*AC008782.1, SLC25A46*
			*rs6894249-G*	2 × 10^−11^	*AC116366.3, IRF1-AS1*
			*rs3763309-A*	1 × 10^−18^	*TSBP1-AS1, HLA-DRA*
Daya M et al., 2019 GCST007266 [17]	5136 Hispanic or Latin American	-	*rs13277810-T*	3 × 10^−8^	*AC246817.2*
			*rs114647118-C*	3 × 10^−7^	*TATDN1*
	9518 African American or Afro-Caribbean		*rs3122929-T*	9 × 10^−7^	*STAT6*
			*rs10519067-G*	2 × 10^−7^	*RORA*
			*rs907092-G*	4 × 10^−12^	*IKZF3*
			*chr6:145053377-C*	8 × 10^−7^	*-*
			*chr4:130040437-T*	2 × 10^−7^	*-*
			*chr18:42607527-G*	8 × 10^−7^	*-*
			*chr19:54192692-G*	5 × 10^−7^	*-*
			*chr1:234597720-A*	9 × 10^−7^	*-*
			*chr17:38040119-C*	3 × 10^−11^	*-*

**Table 2:** Results of miR studies in association with asthma risk.

miRNA	Author	Role in Asthma	Levels in Asthma	Mice/Human	Biofluid/Tissue
miR221	Qin et al., 2012 [82]	induces airway inflammation	increased	mice	BALF/lung tissue
		induces cytokine production	-	-	-
	Mayoral et al. 2011	mast cell degranulation	increased	mice	bone marrow mast cells
	[83]	mast cell cytokine production	-	-	-
miR21	Wu et al., 2014 [74]	positive correlation with IL-13	Increased	human	bronchial epithelial cells
	Lu et al., 2011 [84]	induces Th2 response	-	OVA-mice	-
		eosinophilic accumulation	-	-	-
miR19	Simpson et al., 2014 [85]	promotes IL13 and IL5 production	increased	human	airway T cells
	Haj Salem et al., 2014	targets TGF-b R2 mRNA	increased	human	lung biopsy
	[86]	contributes to airway remodeling	-	-	-
miR145	Collison et al., 2011 [87]	HDM exposure increases miR145 levels involved in allergic inflammation	-	HDM-sensitized mice	airway tissue
miR146	Comer et al., 2014 [88]	anti-inflammatory	increased	human	human ASM cells
		negatively regulates IL1β and COX2	-	-	-
miR126	Mattes et al., 2009 [89]	anti-mir126 reduces Th2 cytokines	-	HDM-sensitized mice	lung tissue and lymph nodes
	Wu et al., 2014 [74]	increases AHR and immune cell migration	increased	human	bronchial epithelial cells

## Data Availability

Not applicable.
